# Skeptical Look at the Clinical Implication of Metabolic Syndrome in Childhood Obesity

**DOI:** 10.3390/children10040735

**Published:** 2023-04-17

**Authors:** Malgorzata Wasniewska, Giorgia Pepe, Tommaso Aversa, Simonetta Bellone, Luisa de Sanctis, Procolo Di Bonito, Maria Felicia Faienza, Nicola Improda, Maria Rosaria Licenziati, Claudio Maffeis, Alice Maguolo, Giuseppina Patti, Barbara Predieri, Mariacarolina Salerno, Stefano Stagi, Maria Elisabeth Street, Giuliana Valerio, Domenico Corica, Valeria Calcaterra

**Affiliations:** 1 Division of Pediatrics, Department of Human Pathology of Adulthood and Childhood, University of Messina, 98121 Messina, Italy; 2 Division of Pediatrics, Department of Health Sciences, University of Piemonte Orientale, 28100 Novara, Italy; 3 Department of Public Health and Pediatric Sciences, University of Torino, 10126 Turin, Italy; 4 Department of Internal Medicine, “Santa Maria delle Grazie” Hospital, 80078 Pozzuoli, Italy; 5 Department of Precision and Regenerative Medicine and Ionian Area, University of Bari “Aldo Moro”, 70124 Bari, Italy; 6 Neuro-Endocrine Diseases and Obesity Unit, Department of Neurosciences, Santobono-Pausilipon Children’s Hospital, 80122 Napoli, Italy; 7 Department of Surgery, Dentistry, Pediatrics and Gynecology, Section of Pediatric Diabetes and Metabolism, University and Azienda Ospedaliera Universitaria Integrata of Verona, 37126 Verona, Italy; 8 Department of Pediatrics, IRCCS Istituto Giannina Gaslini, University of Genova, 16128 Genova, Italy; 9 Department of Medical and Surgical Sciences of the Mother, Children and Adults, Pediatric Unit, University of Modena and Reggio Emilia, Largo del Pozzo, 71, 41124 Modena, Italy; 10 Pediatric Endocrinology Unit, Department of Translational Medical Sciences, University of Naples Federico II, 80131 Naples, Italy; 11 Health Sciences Department, University of Florence and Meyer Children’s Hospital IRCCS, 50139 Florence, Italy; 12 Unit of Paediatrics, Department of Medicine and Surgery, University of Parma, Via Gramsci, 14, 43126 Parma, Italy; 13 Department of Movement Sciences and Wellbeing, University of Napoli “Parthenope”, 80133 Napoli, Italy; 14 Department of Pediatrics, “Vittore Buzzi” Children’s Hospital, 20157 Milano, Italy

**Keywords:** metabolic syndrome, childhood obesity, childhood overweight, cardiovascular risk, hypertension, hyperinsulinemia, dyslipidemia

## Abstract

Metabolic syndrome (MetS) is defined by a cluster of several cardio-metabolic risk factors, specifically visceral obesity, hypertension, dyslipidemia, and impaired glucose metabolism, which together increase risks of developing future cardiovascular disease (CVD) and type 2 diabetes mellitus (T2D). This article is a narrative review of the literature and a summary of the main observations, conclusions, and perspectives raised in the literature and the study projects of the Working Group of Childhood Obesity (WGChO) of the Italian Society of Paediatric Endocrinology and Diabetology (ISPED) on MetS in childhood obesity. Although there is an agreement on the distinctive features of MetS, no international diagnostic criteria in a pediatric population exist. Moreover, to date, the prevalence of MetS in childhood is not certain and thus the true value of diagnosis of MetS in youth as well as its clinical implications, is unclear. The aim of this narrative review is to summarize the pathogenesis and current role of MetS in children and adolescents with particular reference to applicability in clinical practice in childhood obesity.

## 1. Introduction 

Metabolic syndrome (MetS) is defined by a cluster of several cardio-metabolic risk factors, specifically visceral obesity, hypertension, dyslipidemia, and impaired glucose metabolism, which together increase the risks of developing future cardiovascular disease (CVD) and type 2 diabetes mellitus (T2D) [1,2,3]. The early diagnosis of MetS in children and adolescents could enable pediatricians to detect subjects with an increased risk of adverse outcomes later in life and treat them promptly, providing a chance to test the efficacy of early treatment on the incidence of CVD and T2D in adulthood [2]. In fact, obesity in childhood increases the risk of obesity in adulthood by five-fold and correlates with future CVD and T2D [3].

Several classifications have been proposed to define pediatric MetS in the literature. Most are based on the MetS classifications used for adults, proposed by the National Cholesterol Education Program Adult Treatment Panel III (NCEP-ATPIII). However, the use of age- and sex-specific percentiles and pediatric thresholds are mandatory in pediatrics. Taking these aspects into account, subsequently, several classifications introduced cut-offs of age- and sex-specific percentiles and pediatric thresholds modifying the NCEP criteria. All of these consider four main variables: obesity condition, dyslipidemia, glucose metabolism, and blood pressure (BP); however, they differ in the reference values and cut-offs used [1,2,3].

The early diagnosis of MetS in children and adolescents could enable pediatricians to detect subjects with an increased risk of future complications and treat them promptly, offering a chance to test the efficacy of early intervention regarding the incidence of CVD and T2D in adulthood.

The absence of a clear definition of MetS and of specific cut-off values makes it difficult to establish a clear prevalence of the condition and to precisely understand its clinical implication in children and adolescents [1,2,3].

Although there is agreement on the distinctive features of MetS, no international diagnostic criteria in a pediatric population exist. To date, the scientific literature reports many (over 40) definitions of MetS in children [2]. Each of them has different sets of cut-off values and, even when applied to the same population, the estimated prevalence of MetS is different. This important limitation is due to either the sometimes-forced adaptation of the adult definition or the arbitrary cut-off value selection. Moreover, they often do not consider the critical changes in insulin resistance (IR) and body composition during the peri-pubertal period.

As a consequence, the prevalence of MetS in childhood is still not definite and thus the true value of diagnosis of Mets in youth is unclear [2].

This article is a narrative review of the literature and a summary of the main observations, conclusions, and perspectives raised in the literature and the study projects of the Working Group of Childhood Obesity (WGChO) of the Italian Society of Paediatric Endocrinology and Diabetology (ISPED) on MetS in childhood obesity. The aim of this narrative review is to summarize the pathogenesis and current role of MetS in children and adolescents with particular reference to applicability in clinical practice in childhood obesity.

In particular, ISPED aims to promote pediatric studies in the field pediatric endocrinology and diabetes and their diffusion for the ever better physical, mental, and social health of children and adolescents. Moreover, ISPED supports the initiatives aimed at setting up working groups on the scientific, welfare, and health policy topics of endocrinological, diabetological, and pediatric interest for the purpose of developing study and work protocols, starting polycentric research essential to achieve a scientifically valid result, drawing up guidelines on the different topics, and identifying standards of care.

All the authors of this narrative review are part of the Working Group of Childhood Obesity (WGChO) of the Italian Society of Paediatric Endocrinology and Diabetology (ISPED).

## 2. Materials and Methods

The authors focused their search on the usefulness and application of MetS determination in the diagnostic and therapeutic path of obesity in childhood in the context of existing literature data.

The authors performed a literature search in PubMed and EMBASE, using selected key words (‘Metabolic syndrome OR MetS’) AND (‘children OR adolescent’) AND (‘diagnosis OR definition’) AND (‘obesity OR overweight’). Besides the automated search, a manual search for additional relevant publications was carried out in the bibliographies of the papers automatically identified.

All authors independently identified the most relevant papers published in English in the past 15 years, including original papers, metanalysis, clinical trials, and reviews. Case reports, series, and letters were excluded.

The contributions were critically reviewed and collected by all the authors, all of whom approved the final version.

## 3. Results and Discussion

### 3.1. Phenotypes in Pediatric Obesity

Children with obesity who have a favorable metabolic profile with normal glucose metabolism, normal lipids, and normal BP have been described as metabolically healthy obese (MHO) individuals. Currently, there are no universally accepted criteria to recognize children with an MHO phenotype, despite several definitions and cut-off values related to insulin sensitivity and metabolic syndrome components [4,5,6]. In accordance with a recent consensus-based definition of an MHO phenotype [7], most of the cut-off values were adapted from the definition of MetS in children provided by the International Diabetes Federation (IDF) [8], and therefore should facilitate the definition of MHO children. The MHO phenotype is associated with significantly lower body mass index (BMI), lower waist circumference (WC), lower serum uric acid, higher serum adiponectin, and higher serum apolipoprotein A-1 levels than metabolically unhealthy obese (MUO) individuals [9].

Furthermore, the MHO phenotype is more common among younger and prepubertal children than the MUO phenotype [10,11,12]. These observations may be explained by the changes that physiologically occur during puberty, including a decrease in insulin sensitivity. Thus, MHO children may become a MUO phenotype during pubertal development [13].

Hyperuricemia has been related to the increased risk of developing MetS, T2D, and CVD in children with obesity [14]. Low apolipoprotein A-1 serum levels are linked to endothelial dysfunction in children with obesity [15]. Several studies evaluated the association of MHO phenotype and the risk of subclinical atherosclerosis [16,17]. Lin et al. demonstrated that during a follow-up of 4.4 years, 46.8% of MHO subjects developed a metabolically unhealthy status, and an increased risk of subclinical atherosclerosis [17]. The relationship between BMI and subclinical atherosclerosis was partially mediated by BP and glucose levels.

A prospective cohort study demonstrated that both MHO and metabolically unhealthy normal weight (MUNW) groups are associated with an increased risk of endothelial dysfunction [18], while the Bogalusa Heart Study reported that no difference in adulthood carotid intima-media thickness (CIMT) was found between MHO children and their metabolically unhealthy counterparts [19]. In a cohort study of 1220 children and adolescents, CIMT values showed a significantly increasing trend from metabolically healthy normal weight (MHNW) to the MUO group. The results of this study demonstrated that normal weight children, even with an unhealthy metabolic profile, did not show a higher risk of high CIMT. Likewise, obese children with healthy metabolic status are not at higher risk [20]. In another comparative evaluation between children and adolescents with MHO and MUO, subjects with MUO not only had a significantly higher CIMT but also had significantly greater left ventricular dimensions, epicardial adipose tissue, ascending aorta diameter, carotid, and ascending aorta stiffness as well as a significantly more impaired global longitudinal strain compared to MHO patients [21]. Non-alcoholic fatty liver disease (NAFLD) has been associated with obesity, and an alanine aminotransferase (ALT) test is the best screening tool to enable the diagnosis of NAFLD in children [22]. Obese children with MetS are more likely to have advanced liver fibrosis compared to those without MetS [23].

Studies have demonstrated that adults with the MHO phenotype have a greater risk of developing chronic kidney disease (CKD) with respect to their normal weight peers [24,25].

However, data on the glomerular filtration rate (GFR) are discordant. Arora et al. did not find any significant difference in the serum creatinine and estimated GFR (eGFR) between MHO and MUO children, although 72.2% of schoolchildren with obesity were at increased risk of developing CKD [26].

Recently, a meta-analysis commissioned by the World Health Organization (WHO), encompassing 159 studies, has been published, demonstrating that breastfeeding protects against overweight and obesity. Age population included children under 9 years of age in 73% of cases and more than half of the studies evaluated at least 1500 participants, controlling for the number of months of breastfeeding. In high-income countries, the benefit of breastfeeding was demonstrated, adjusted for socioeconomic status, birth condition, and maternal anthropometry. In the total population (including middle or low-income country), the benefit of breastfeeding was stronger in the pediatric population compared to adults. Moreover, in studies with fewer confounding factors, the benefits of breastfeeding were demonstrated [27].

These studies in the field of metabolomics and childhood obesity will be of great importance regarding the development of more personalized prevention and treatment in the future.

Thus, the distinction between obese phenotypes was able to provide more effective and targeted therapies for children and adolescents with obesity.

### 3.2. Origins of MetS

At a postnatal age, rapid and excessive weight gain in early life could be a risk factor for the development of MetS [28]. Several authors have investigated the importance of postnatal growth, linking infants with higher BMI or rapid weight increase with an enhanced probability of obesity in later life and the risk of metabolic alterations [29,30].

Nutrition is an important factor in infant growth from the first months of life. Numerous studies in humans investigated the impact of breastfeeding versus formula-feeding nutrition. Overall, studies indicate that breastfeeding exerts a protective role in the development of overweight and obesity during childhood [31,32,33]. Despite studies being affected by confounding factors, such as the observational study design, the time, duration, exclusivity of breastfeeding, or the study location, most have demonstrated the positive effect of breastfeeding, which is more evident related to the duration of breastfeeding for more than 4–6 months and its exclusivity [34,35]. Recently, a meta-analysis commissioned by the World Health Organization (WHO), including 159 studies, has been published demonstrating that breastfeeding protects against overweight and obesity. The population included children under 9 years of age in 73% of cases, and more than half of the studies evaluated at least 1500 participants, controlling for the number of months of breastfeeding. In high-income countries, a benefit of breastfeeding was demonstrated adjusted for socioeconomic status, birth condition, and maternal anthropometry. In the total population (including middle or low-income countries), the benefit of breastfeeding was stronger in the pediatric population compared to adults. Anyway, in studies with fewer confounding factors, the benefits of breastfeeding were demonstrated [36].

The mechanism linking breast or artificial feeding with obesity may be found in the different growth patterns: breastfed babies grow slowly compared to artificially fed babies. In particular, formula-fed babies increase weight in the first weeks of life, while breastfed infants lose weight, determining different growth channels [30], as they are different in energy and lipid and protein content. A higher protein and energy intake at a young age may influence the growth curve, facilitating the development of obesity and metabolic derangements [31], determining more rapid weight gain, fat deposition, reducing insulin sensitivity, upregulating insulin, and IGF-1 production [30]. Instead, several studies demonstrated that breastfeeding reduces the risk of metabolic alterations, lowering insulin levels [37], lipid profiles [38,39], and the risk of the development of T2D [36,39,40].

Thus, breastfeeding should be recommended as a natural behavior that allows healthy growth, thus preventing overweight and consequent metabolic alterations in later life.

In August 2020, the WHO changed its recommendations, suggesting exclusive breastfeeding for the first 6 months of life (instead of 4 months) and then from six months up to 2 years of age with the addition of complementary foods [41].

Reviewing the literature, most data showed that, among formula-fed infants but not breastfed infants, the introduction of solid foods before 4 months of age is associated with the increased prevalence of obesity at 3 years of age [30,31,42]. Verga et al. conducted a recent systematic review investigating the effects of the time of introduction of solid food on growth at 1 year of age and on the development of obesity at 3–6 years of age. They concluded that the introduction of complementary food at 4 or 6 months does not influence growth at 12 months of life or on the percentage of obesity at 3 and 6 years. Therefore, introducing complementary foods before the age of 6 months in healthy-term infants does not present any beneficial effect, determining, on the contrary, a reduction in human milk feeding [43].

Moreover, some data indicate that the quality of feeding also influences infant growth and the risk of developing obesity in later life [30,44,45]. In particular, a balanced diet, rich in fiber and poor in fat, could be proposed, particularly in formula-fed babies [45].

### 3.3. Endocrine Disrupting Chemicals

Humans are exposed to several endocrine-disrupting chemicals (EDCs), which may interfere with endocrine system functions through the specific pathogenic mechanisms of action [46,47]. Our knowledge of EDCs effects on human health has greatly improved over the last decade [48,49,50]. “Obesogen” EDCs accumulate in adipose tissue, and this can lead to interactions and changes in the endocrine activity of adipose tissue and homeostatic systems underlying weight control.

The mechanisms of action currently known to be involved with the action of EDCs as metabolic disruptors and obesogens are multiple (currently 56). These include effects on nuclear receptors, such as aryl hydrocarbon and retinoid X receptors, aside from PPAR γ; the stimulation of estrogen receptors and anti-androgenic effects; interference with adipocytokines; interference with mesenchymal cell differentiation into adipocytes; interference with thyroid hormones; interference with the amount of white and brown adipose tissue; changes in microbiota, which in recent years have been shown to be important for metabolism [51,52,53]; changes in oxidative stress [54]; and finally, epigenetic effects that are described separately in this review. Any EDCs acting as agonists on peroxisome proliferator-activated receptor-gamma will directly promote adipogenesis, increasing both the number and size of fat cells [48,55]. Furthermore, “diabetogen” EDCs can disrupt β-cell function, causing IR and defects in insulin production and secretion [56].

Contact with EDCs occurs through contact, ingestion, and inhalation. The main classes of known obesogenic man-made chemical substances are contained in industrial solvents and lubricants and their bioproducts, in personal care products and in food containers, in textiles and clothing, in medical tubing, in children’s products, in anti-bacterial agents and pesticides, in electronic devices, and in building materials.

The main products in each class and their effects in the relationship between metabolism and obesity are reported hereafter.

Persistent organic pollutants (POPs), including some pesticides, such as DDT and derivatives and polychlorinated biphenyl(PCB) compounds and some non-POPs, are well known “obesogens” and “metabolic disruptors”. POPs are lipophilic and accumulate in the fat mass of living organisms because of their resistance to biodegradation, so they are able to alter metabolic functions, leading to overweight and the development of obesity [57]. Non-POPs are also suggested to favor the increase in weight mass and alter carbohydrate and lipid homeostasis [58,59]. In utero, early postnatal life, and/or pubertal development are periods which are highly susceptible to EDCs exposure. The disruption of metabolic systems during these critical periods impacts the development of non-communicable metabolic diseases, such as obesity, T2D, and MetS [60,61].

Exposure to bisphenol A (BPA) during perinatal and postnatal periods increases the risk of developing obesity. A prospective study investigated 719 mother–child pairs and showed a relationship between the gestational levels of BPA and central adiposity in early childhood [62]. A further study confirmed that BPA exposure, measured in urine samples from 298 boys aged 9–11 years, was associated with a greater risk of central obesity [63]. Lastly, a positive association of dietary exposure to BPA and total bisphenols with being overweight/obese was found in Spanish adolescent girls [64]. Associations between maternal prenatal BPA exposure and child cardiometabolic risk factors at 2 years of age were evaluated in a prospective cohort involving 218 pregnant women from China. BPA exposure during the prenatal period was associated with increased BP in girls and blood glucose in boys [65]. Again, obese children with MetS were found to have significantly higher urinary BPA levels than obese children without MetS and in both obese groups levels were higher than in healthy controls [66]. Bisphenols may also affect uric acid metabolism, and this aspect is of concern, as a potential relationship between hyperuricemia and hypertension, MetS, fatty liver disease, and cardiovascular disease in pediatric populations has been identified [67,68,69]. The associations between BPA, bisphenol S (BPS), and bisphenol F (BPF) exposure and serum uric acid concentrations were evaluated in the Environment and Development of Children cohort study, encompassing 489 six-year-old Korean children. In boys, urinary BPS levels were significantly and positively associated with serum uric acid concentrations—the higher the BPS levels, the higher the serum uric acid concentrations [70].

Obesity in children was reported to be induced by phthalate exposure during early life (in utero and childhood). Gender-specific and trimester-specific relationships between bis-2-ethylhexyl phthalate (DEHP) exposure and birth offspring growth data (BMI and weight gain rates) were longitudinally confirmed at 6, 12, and 24 months in the prospective cohort study from China [71]. In the CHAMACOS study, the levels of 11 phthalate metabolites in the urine of 345 mothers during pregnancy were analyzed along with their children’s height, weight, WC, and percentage of body fat. In this 12-year-old population, the in utero levels of diethyl phthalate, dibutyl phthalate, and DEHP metabolites were positively associated with overweight or obesity [72]. Recent results from the same study suggested that higher prenatal exposures to the cumulative biomarker mixture (phthalates and parabens) trended with a greater risk for obesity in early childhood [73]. A study from China, involving 789 children aged 7–10 years, demonstrated that the detection of urinary phthalate metabolites was common in recruited subjects and exposure level was associated with risk of abdominal obesity. Compared with the Q1 group of phthalate metabolites, the risk of childhood abdominal obesity increased to 429% and 273% for the Q4 group of exposure to mono-ethyl phthalate (MEP) and mono-iso-butyl phthalate, respectively [74]. Studies revealed controversial results regarding the association of exposure to phthalates with cardiometabolic risk factors in children and adolescents. A systematic review and meta-analysis, encompassing 17 cohorts and 15 cross-sectional and 3 case–control studies, observed a significant association between phthalates and their metabolite concentrations with BMI, BMI z-score, WC, low-density lipoprotein cholesterol, triglyceride, and glycemia [75]. Recently, a study from China, involving 829 children, demonstrated that phthalate exposure during childhood might significantly increase the risk of dyslipidemia and higher levels of lipid profiles, particularly in boys [76].

In utero exposure to dichlorodiphenyltrichloroethane (p,p’-DDT), dichlorodiphenyldichloroethylene (p,p’-DDE), and hexachlorobenzene (HCB) may increase the risk for rapid weight gain in infancy [77] and high BMI later in childhood [78]. Longitudinal positive associations between prenatal exposure to p,p’-DDE and p,p’-DDT with other obesity-related outcomes were also reported in children [79]. The association between in utero POP exposure and major risk factors for cardiometabolic syndrome in adulthood was recently reported in a longitudinal study involving 379 children from Spain, ranging from 4 to 18 years of age. HCB exposure in the 3rd-tertile was associated with higher BMI and weight-to-height ratio z-score, and a continuous increase in HCB levels was associated with higher body fat %, systolic and diastolic BP z-score, cardiometabolic-risk score, and lipid biomarkers [80].

Positive associations were found between maternal serum perfluorooctanesulfonic acid (PFOS) and perfluorooctanoic acid (PFOA) concentrations from 412 pregnant Norwegian and Swedish women and child overweight/obesity. In children at a 5-year follow-up, both the BMI-for-age-and-sex z-score and the triceps skinfold z-score were found to increase per logarithmic-unit increase in maternal serum PFOS and PFOA concentrations [81]. Furthermore, exposure to these perfluorinated compounds increased the risk for CVD [82], dyslipidemia, and T2D [83].

To date, few studies have analyzed the role of EDCs mixture exposure in the etiology of obesity and dyslipidemia. The Korean National Environmental Health Survey 2015–2017 cross-sectional study quantified the urinary concentrations of eight phthalate metabolites, three phenols, three parabens, and one pyrethroid pesticide metabolite in children, adolescents, and adults. EDCs mixtures were associated with higher BMI in adolescents and a higher triglyceride/high-density lipoprotein cholesterol in male adolescents [84].

In conclusion, much scientific evidence indicates that exposure to EDCs during the prenatal, early infancy, and/or pubertal periods may cause the abnormal distribution of adipose tissue, its excess, and subsequent metabolic complications. We need more conclusive data on the relationship between EDCs and metabolism, but strategies to reduce EDC exposure from early life stages may be necessary to reduce the risk of future metabolic diseases [60].

### 3.4. Heterogeneity of MetS Definitions in the Pediatric Age Group

According to the definition of MetS used, the prevalence of the condition also differs among the same population [2]. Today, an obesity epidemic is occurring—specifically, pediatric and adolescent obesity rates have increased from less than 1% in 1975 to nearly 6% in girls and almost 8% in boys in 2016 [3]. Consequently, comparing pediatric MetS prevalence is made challenging, partly by the changing epidemiology of childhood obesity, partly by using different diagnostic cut-offs, and partly by the enrolment of young people with varying nutritional status [2]. All these factors represent variables that do not allow an accurate estimate of the prevalence of MetS in childhood [2]. An additional element not to be forgotten related to MetS in adolescence is puberty, a period which is physiologically characterized by reduced insulin sensitivity that spontaneously returns to normal ranges at the end of puberty [2,85]. In particular, some studies have shown a worsening of cardiovascular risk factors (hypertension, dyslipidemia, impaired glucose metabolism) at the beginning of puberty, which spontaneously improves at the end of this period, regardless of body weight modifications [2,13].

As previously mentioned, diverse classifications have been suggested to define pediatric MetS, but they all focus on four principal variables: obesity, dyslipidemia, glucose metabolism, and BP [2].

Concerning obesity, the vast majority of studies consider WC as a parameter [86,87,88,89,90,91,92], taking a percentile ≥ 90° [87,88,89,91,93,94] as the reference value, which is age-, sex-, and ethnicity-specific in some cases [87,91,94,95]. In a few cases, obesity is assessed by BMI [96,97,98], for which either percentile or z-score is considered. It is well-known that visceral obesity plays a key role in the pathogenesis of MetS. Although BMI is a widely used index as a predictor of MetS, it cannot provide information on fat distribution. It would be more appropriate to use an anthropometric index more closely associated with central obesity, such as WC [99,100]. Limitations for using this include the absence of a reference point for WC (some authors use the midpoint between the last rib and the top of the iliac crest, while others utilize the upper edge of the iliac crest or the level of the umbilicus); ethnic difference in visceral adipose tissue (VAT) (Asian Indians have more VAT, despite having a lower body mass than white Europeans, and young white individuals have more VAT than young African Americans at the same BMI) [101], and body shape during puberty (boys develop a more android shape by depositing more fat in the abdomen, while girls deposit it in the hips and limbs, forming a gynoid shape). Based on these considerations, it is important to use population-, sex-, and age-specific WC cut-offs to identify the cardio-metabolic risk associated with weight gain. The waist-to-height ratio (WHtR), calculated by dividing WC by height, has several advantages over BMI or WC. First, a WHtR value of 0.5 is suggested as a cut-off for abdominal obesity and health risks in children and adults, without differences of sex, ethnicity, and age, so it does not require reference percentiles for diagnosis. Moreover, a recent meta-analysis of children [102] showed that WHtR is comparable to both WC and BMI in terms of screening power for cardio-metabolic risk; however, to date, WHtR is not usually considered in MetS classifications.

IR is another crucial component of MetS and is a player in the development and progression of cardiometabolic risk factors, being related to hypertension, dyslipidemia, and T2D [103]. IR is a reduced tissue response to the action of insulin and is associated with excess adipose tissue, especially VAT. In pediatric age, IR is a physiological condition that promotes body growth, reaching its peak at puberty and then declining to prepubertal values. IR also correlates with ethnicity: African American, Hispanic, Pima Indian, and Asian children are less sensitive to insulin than Caucasian children. In terms of diagnosis, homeostatic model assessment (HOMA)-IR is a widely used tool based on the relationship between fasting glucose and insulin levels; however, it is little used in the pediatric population as a reliable reference range is not yet available in this population group [103]. Therefore, without strong surrogate biomarkers of specific IR, the Insulin Resistance Consensus Group [104] does not suggest IR screening in the clinical setting for children. In contrast, the American Diabetes Association (ADA) [105] recommends risk-based screening (fasting plasma glucose (FG), an oral glucose tolerance test which tests impaired glucose tolerance (IGT) and glycated hemoglobin) for prediabetes and/or T2D in children with overweight, obesity, or additional risk factors. Impaired FG (IFG) is the most frequently used parameter in MetS classifications, and the IDEFICS Study also considers HOMA-IR to be appropriate [91].

Obesity is typically associated with an ongoing dyslipidemia, consisting of high levels of total cholesterol, LDL cholesterol (LDL-C), and triglycerides (TG) and low levels of HDL cholesterol (HDL-C). The identification of lipid abnormalities is crucial for the prevention of future CVD. The proposed MetS definitions have different absolute cut-off values [87,88,89,92,93,97] or percentiles [87,90,91,94,96], either age-, sex-, and/or ethnicity-specific or nonspecific [98], extrapolated from studies or identified by the authors. The Expert Panel on Integrated Guidelines for Cardiovascular Health and Risk reduction in Children and Adolescents [106] proposes certain absolute values as diagnostic cut-off points: in this case, LDL-C ≥ 130 mg/dL and TG ≥ 100 mg/dL in children aged 0–9 years and TG ≥ 130 mg/dL in those aged 10–19 years, at HDL-C < 40mg/dL. The panel recognizes non-HDL cholesterol level (calculated by subtracting HDL-C from plasma total cholesterol level) as a more predictive index of persistent dyslipidemia than total cholesterol or LDL-C or HDL-C levels alone. However, in the MetS classifications, HDL-C and TG are usually considered to detect dyslipidemia.

The fourth element of MetS is hypertension, where early treatment is fundamental in order to reduce future CVD [107]. The BP levels suggested by the proposed definitions of MetS are widely heterogenous (Table 1).

Flynn et al. [27] recently published a guideline on pediatric hypertension, which is an update of the 2004 Fourth Report on the Diagnosis, Evaluation and Treatment of High Blood Pressure in Children and Adolescents; this guideline defines “elevated BP” as a BP value ≥ 90th percentile to < 95th percentile in children aged 1–13 years and 120/<80 mmHg to 129/<80 mmHg in adolescents aged ≥ 13 years.

The majority of pediatric MetS diagnostic criteria, specifically those of the International Diabetes Federation (IDF) [8], those of the IDEFICS study [91], those recommended by Cook et al. [88], and those proposed by De Ferranti et al. [90], take into account the period between 10 and 16 years of age. A new international definition of MetS, proposed by Zong et al. [95] in 2022, is aimed at children and adolescents between the ages of 6 and 17 years (Table 1); the authors used the greatest value to propose a unified international MetS definition.

Despite a variety of definitions suggested to diagnose MetS in children and adolescents in the past two decades, a universally accepted definition of MetS at pediatric age to ensure a clear diagnosis is not yet available [2]. Indeed, an important limitation for the universally accepted definition of MetS in pediatrics is the use, for each criterion, of reference percentiles as cut-off values, which are often not specific to the patient’s nationality/ethnicity [2], are not very practical, and are poorly applicable in the clinical setting.

To overcome these challenges, the IDF proposed to assess the prevalence of MetS in childhood and the use of diagnostic criteria measures rapidly accessible in clinical practice, without the adoption of multiple reference tables; the limits remain the use of the modified form of the adult criteria [2,8].

In summary, there is still the need for an internationally accepted definition of pediatric MetS that could more easily allow preventive and therapeutic measures. Moreover, proposals for a MetS classification for younger children, aged 2 to 5 years, are also necessary, considering that risk factors are already present in early childhood [108].

### 3.5. Epidemiology of MetS

The true prevalence of MetS in childhood and adolescence is still a critical, open issue, due to the lack of consistent and uniformly accepted diagnostic criteria for MetS. A large number of studies have investigated and reported a different prevalence of MetS, based on different populations by size, age, gender, and ethnicity; different definitions of obesity and of MetS; different methodological approaches in study settings; and different types of surveys [8,109]. The prevalence of MetS thus differs and study results are often not comparable.

Two recent systematic reviews report data on the prevalence of MetS in a wide population of children and adolescents. They considered MetS in conjunction with the four most recognized and used diagnostic criteria in childhood (IDF, modified ATP-III) [88,90], without any restriction on the setting. In both reviews, the different prevalence for geographical region and country’s development status are reported. Table 1 shows the pooled data emerging from the two systematic reviews.

According to both reviews, IDF is the most used classification system, followed by modified ATP III [88,90].

Due to the different methodologies used, the median prevalence for predefined subgroups was indicated. MetS among the overweight/obese (OW/OB) population of 3906 children and adolescents belonging to medium/low income countries (MLIC) had a pooled prevalence of 24.09%, 36.5%, and 56.32%, using IDF, ATP III and de Ferranti criteria, respectively; among 45,889 OB children and adolescents, the prevalence of MetS showed a median value of 26.1%, according to IDF criteria, and ranged from 26.3 to 40% in Chile and Mexico, respectively, according to Cook’s criteria.

Data concerning the different geographical areas considered in the two reviews indicate large disparities in the prevalence of childhood MetS between countries, which were mostly represented by MLIC. Indeed, very few data were provided for high-income countries (HIC) and no studies from the United States (US) population were included at all. This finding might be explained by the fact that while the review by Bitew et al. was specifically addressed to analyze the prevalence of MetS among youths from MLIC [110], the review by Obita et al. had the definition of OB according to WHO as a selection criterion, a classification system which is not used in the US [111].

A recent survey on a representative sample of 2325 teens with OW/OB, aged 12–19 years, from the 1999–2018 National Health and Nutrition Examination Survey cohort, indicated a median prevalence of MetS of 2.56% (ranges 1.65–3.96%) and 20.09% (ranges 16.96–23.61%), respectively, in OW and OB, using the IDF definition, with differences in prevalence among population subgroups [112].

The pooled prevalence of MetS computed for the different genders shows higher values for males than females both in the paper by Obita et al. (35% vs. 12.9%) and in the paper by Bitew et al. (24.0% vs. 6.6%), confirming data indicated by previous studies [110,111]. However, these data refer to a small population of the entire cohort; consistent data on gender disparity are still lacking also for the US population.

The two systematic reviews and the recent US survey, which include a very high number of OW/OB children and adolescents from different countries around the world, are valuable for assessing the current global trend of MetS in childhood. However, when evaluating the results from different population-based studies, the proportion of children being affected by the MetS might contain considerable dispersion, attributable to different factors, such as prevalence and severity of weight excess, age range, sex distribution, ethnicity, lifestyle, environment, and socio-economic status.

Indeed, the reported pooled data from the two reviews and the study from the US have several limitations. First, they refer to different populations of OW/OB and OB children and adolescents, not entirely overlapping in age, from different countries, both from LMIC only or LMIC and HIC combined. Second, the countries that were analyzed as a whole do not fully reflect the situation of the entire continent, and every cohort of the included studies might not be representative for the overall country population. Gender differences were evaluated in only a few cases of the entire population in order to achieve conclusive real data.

The pooled prevalence of MetS, according to different definitions from the two most recent systematic reviews, are presented in Table 2.

In conclusion, alongside the lack of a unanimous definition of MetS, to define the real prevalence of MetS, robust and globally distributed data, conducted on homogeneously recruited and investigated populations, from representative countries of each geographical area, are still lacking.

It is likely that the definition of the prevalence of Mets could be a truly complex phenomenon that must take into account age, gender, ethnicity, food habits, and lifestyle specificity to avoid simplistic conclusions, which can lead to the underrepresentation of the growing health burdens and complications of obesity in childhood.

### 3.6. Factors Changing Epigenetics and Predisposing to MetS

It is well known that most of the effects mediated by EDCs are due to epigenetic changes occurring during in utero exposure and possibly during the first year of life mediated by exposure through breast milk. These changes mainly comprise differences in DNA methylation and histone acetylation, keeping in mind that ubiquitination, methylation, and phosphorylation are also implicated in the control of DNA transcription, as well as non-coding RNA—in particular, miRNA networks are all mechanisms of gene regulation that can be affected subsequently modifying gene expression.

Interestingly, it has been shown that miRNA expression changes in the placenta depending on different conditions, such as pre-eclampsia, gestational diabetes, small for gestational age (SGA), ectopic pregnancy, and premature delivery [113]. It should be remembered that approximately 10% of subjects born SGA develop metabolic syndromes in later life, largely due to the reprogramming of metabolic and endocrine pathways during intrauterine growth and through their relationship with a rapid catch-up growth in early life [114].

As pointed out above, postnatal nutrition also plays an important role. The duration of breastfeeding has been shown to be related to both BMI and DNA methylation, with a shorter period of breastfeeding being associated with an earlier and greater increase in BMI, and patterns of DNA methylation differ according to the duration of breastfeeding [115]. Interestingly, mothers’ features, such as BP, have been shown to affect DNA methylation and cell types within the placenta [116], subsequently affecting the predisposition toward disease in later life. Indeed, breast milk has been shown to be an excellent matrix to study environmental exposure [114,115,116,117,118,119,120,121].

In detail, for example, one study detected 31 organic contaminants and 14 toxic and essential elements in breast milk samples from a Spanish cohort of nursing mothers, stored in a biobank [122]. Some contaminants were found to be higher in breast milk samples from low-income mothers, such as dichlorodiphenyltrichloroethane (DDT) and dichlorodiphenyldichloroethylene (DDE), and differences were also seen in primiparous mothers compared with multiparous [122]. Higher levels of bisphenol A (BPA) in low-income pregnant US women have been described and have been associated with adverse effects on offspring [123]. A US study detected up to 172 different chemicals in breast milk samples, most of which are not yet subject to regular monitoring [124], and a further Spanish study detected at least one contaminant in breastmilk among BPA, benzophenones, and parabens [125].

Maternal metabolic features are also well known to influence the metabolic outcome of offspring both in animals and in humans [126] and effects have been described to be transgenerational, generally up to two generations, with epigenetic changes being observed in both oocytes and sperm [127,128,129]. The Avon longitudinal study of parents and children seemed to identify a stronger association between changes in DNA methylation in offspring and the mother’s obesity than with paternal obesity [130]. Overfeeding during gestation leads to the derangement of glucose metabolism in swine that has been demonstrated at a molecular level. These findings are in line with the human findings above [131].

Many genes are well known to be epigenetically regulated in obesity [132], confirming that any changes in expression are very likely to produce an effect on weight, weight gain, energy expenditure, the regulation of appetite, glucose and fat metabolism, and cardiovascular risk.

A good model of the consequences of obesity, increased glucose and free fatty acid concentrations on offspring is that of gestational diabetes, where the reprogramming of the metabolism in the central nervous system in muscle, heart, adipose tissue, pancreas, liver, and spleen have all been shown in the fetus, leading to increased risk for CVD and negative metabolic changes, in particularly relative to energy expenditure [133].

Concluding, both environmental exposure, in particular to endocrine disruptors, and adverse metabolic conditions in utero predispose to an increased risk of derangements in glucose and lipid metabolism, mainly through epigenetic changes, and increase cardiovascular risk in later life.

### 3.7. Etiopathogenesis of Cardiovascular Disease in Children and Adolescents with Obesity

IR and obesity, particularly when associated with increased visceral adiposity, are two important elements in the pathogenesis of MetS and all related complications [134]. There is a higher incidence of cardiovascular morbidity and mortality in patients with obesity affected by MetS [135]. Increasing evidence supports the role of visceral adiposity in the etiopathogenesis of early cardiovascular risk, independent of the severity of obesity, i.e., BMI, as it is a predictor of CVD and cardiovascular mortality from childhood [136].

The specific storage of VAT reflects more the metabolically pathogenic state of increased adiposity, sometimes referred to as MUO. While the ability of subcutaneous adipose tissue (SAT) to store energy through adipocyte hypertrophy, proliferation, and differentiation is limited, energy overload may occur with an increased supply of circulating fatty acids to body organs that are not dedicated to lipid storage, potentially contributing to the fattening of skeletal muscle, liver, pancreas, and heart, causing IR, inflammation, and all the consequent characteristics of MUO [137]. In addition, the increase in pericardial and epicardial adipose tissue can cause the mechanical impairment of cardiac function through the mechanical constriction of the pericardium, causing impaired left ventricular diastolic disfunction and heart failure with preserved ejection fraction [136,138]. Moreover, fat deposits in the subcutaneous tissues of the upper airway district constrict the airways and decrease muscle activity, resulting in episodes of hypoxia and apnea, which eventually lead to sleep apnea. Obstructive sleep apnea and hypoventilation syndrome (OSAS) is an independent risk factor for CVD in subjects with obesity. It can increase the risk of atherosclerosis, pulmonary hypertension, and left and right ventricular failure [139].

A genetic predisposition, in the case of exposition to high food intake, a sedentary lifestyle, and other environmental risk factors, causes the deposition of excess energy loads in fat deposits other than adipose tissue and triggers adiposopathic endocrine and immune responses, contributing to metabolic disease and increasing the risk of CVD [140]. Adipose tissue dysfunction, i.e., adiposopathy, is the condition that characterizes the unhealthy metabolic phenotype of obesity and has a causal role in the initiation and development of pathophysiological events that result in severe chronic cardio-metabolic diseases, associated with a higher risk of early mortality [137].

Excessive adipocyte hypertrophy may contribute to intracellular hypoxia, which induces adipocyte dysfunction and pro-inflammatory response. This leads to the greater production of pro-inflammatory factors, i.e., tumor necrosis factor alpha and interleukin 6, which can contribute to IR.

An IR condition dose not suppress postprandial gluconeogenesis and reduces peripheral glucose non-oxidative disposal, leading to postprandial hyperglycemia, which may contribute to atherosclerosis via multiple mechanisms. Elevated circulating glucose levels induce endothelial dysfunction mediated by oxidative stress, increased systemic inflammation, the activation of the receptors for advanced glycation end-products (RAGE), increased LDL oxidation, the dysfunction of endothelial nitric oxide synthase (eNOS), and platelet hyperactivity [141]. In turn, hyperglycemia may contribute to IR through glucotoxicity, in a vicious cycle.

In addition, in a pediatric cohort, a significant higher value of AGEs/sRAGE-ratio among overweight/obese children and the expression of a relative shift to oxidant from antioxidant factors was demonstrated, suggesting an AGE/RAGE-related oxidative homeostasis dysregulation that could enhance susceptibility to oxidative/inflammatory tissues damage. The severity of overweight, influencing the increase in oxidative stress in human organisms, especially children, may contribute to the pathogenesis of long-term cardiovascular and metabolic alterations [142].

Adipocyte dysfunction impairs the adipogenic signaling mechanism and fat storage in turn, results in an increase in circulating free fatty acids and lipotoxicity in different organs with potential adverse metabolic consequences, including IR, dyslipidemia, NAFLD, and high BP [137]. High levels of unesterified free fatty acids in circulation increase the hepatic secretion of very low-density lipoprotein, reduce HDL-C levels, and increase small, denser LDL particles that are pro-atherogenic. It is well known that abnormal lipid ratios are powerful predictors of CVD [143]. Inflammation and lipotoxicity induced by adiposopathy increases the likelihood of low-density lipoprotein oxidation, a factor of decisive importance in the pathogenesis of atherosclerosis [144]. Atherogenic lipoproteins can become trapped in the subendothelial space, where they undergo oxidation and removal by arterial macrophages, resulting in endothelial dysfunction, foam cells, fatty streaks, and atherosclerotic plaque formation [144]. Systemic and vascular inflammation are fundamental to all aspects of the atherosclerotic process, from fatty streak development to atherothrombosis. The progressive enlargement of the atherosclerotic plaque can produce the chronic, hemodynamically significant narrowing of the artery, resulting in angina or claudication, and the acute rupture of the plaque can cause myocardial infarction and/or stroke [145].

Obesity-related cardiovascular and metabolic comorbidities, including elevated BP, dyslipidemia, and T2D, have been associated with the extension of atherosclerotic disease. Obesity also leads to higher BP due to the activation of the renin–angiotensin–aldosterone and sympathetic nervous systems [146]. Increased CIMT is considered an early marker of atherosclerosis in adolescents and young adults with obesity [147,148].

The etiology of CVD is due to a synergy of environmental and genetic factors. The Western lifestyle, which is one of the determining environmental factors, is characterized not only by a combination of excessive calorie intake, altered macronutrient ratios and sedentariness but also by an increasingly high intake of ultra-processed foods. Recent evidence supports that ultra-processed foods are obesogenic and detrimental to cardiometabolic health. Relevant to this theory, gut microbiome alteration plays a key role in regulating energy and lipid homeostasis and low-grade systemic inflammation, with potential implications for adiposopathy and cardiometabolic related disease [149]. The correlation between diet, microbiome, and intestinal barrier has been demonstrated through microbiome-mediated metabolism of fiber and accessible dietary carbohydrates, leading to the increased production of short-chain fatty acids (SCFA) and other metabolites, such as trimethylamine-N-oxide (TMAO) and secondary bile acids [150]. These, in turn, modulate the barrier function of the intestinal mucosa and increase its permeability: the passage of endotoxins, including pathogen-associated molecular patterns (PAMPs), such as lipopolysaccharide (LPS), has systemic immune-stimulating effects, contributing to a chronic inflammatory state and the pathogenesis of cardiovascular risk [150,151].

### 3.8. Outcomes and Complications of Childhood Obesity—Association with Cardiometabolic Risk and/or Organ Damage

**Figure 1:**
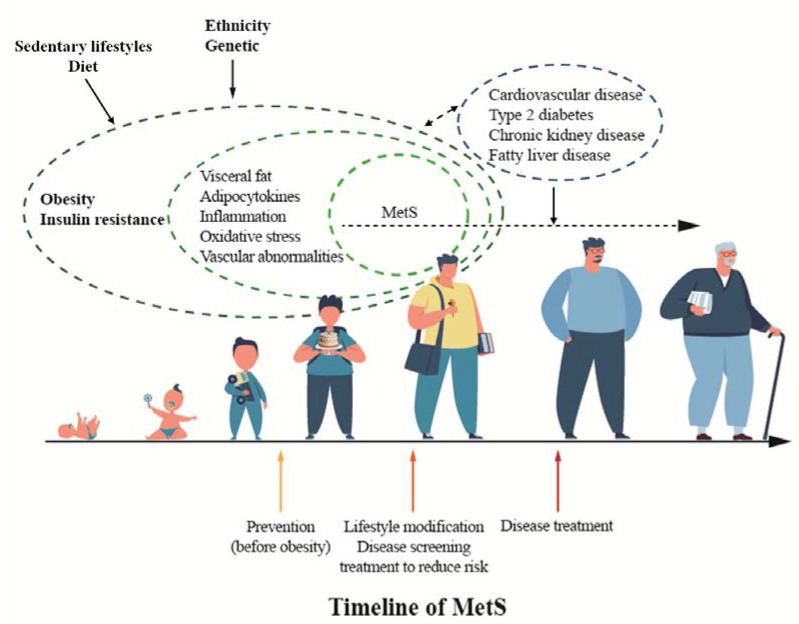
Timeline of metabolic syndrome (MetS).

Obesity is the most important determinant of whole body-reduced insulin sensitivity in childhood [96,152] and the prevalence of MetS is significantly higher among obese children and adolescents, with an incidence and severity that generally increases with a worsening of the weight excess [96]. The pathophysiology of MetS in childhood is not completely understood, although evidence suggests that obesity, IR, and chronic and systemic inflammatory state, in the presence of a genetic predisposition, are the key risk factors for developing the condition [153]. A predisposition to MetS may also originate in intrauterine life, with the main predisposing factors being as follows: maternal obesity, being large or small for gestational age, gestational diabetes, and epigenetic mechanisms (Figure 1) [153].

Given the long-term associations between MetS and future diseases, it is vital to consider and diagnose this condition in obese children and adolescents. This is especially important when we consider that the prevalence of MetS has risen from 3.9% in the 1970s [154] to 9.8% in the last few years [155], meaning that in the current generation of children and adolescents, many more are at risk of developing T2D and CVD in the future [154,156]. In addition, MetS is linked to metabolic-associated fatty liver disease (MAFLD) [157], renal dysfunction with microalbuminuria, and hyperuricemia, which can lead to chronic kidney disease [158,159], obstructive sleep apnea (OSA), polycystic ovary syndrome (PCOS), orthopedic complications, abnormal cytokine and/or adipokine profiles, proinflammatory and prothrombotic tendencies, and mental and emotional health disorders [160,161]. Furthermore, the co-morbidities that are linked to obesity and IR further aggravate metabolic parameters and are detrimental to the general wellbeing of the subject [160,161].

It is therefore essential that practitioners identify at-risk children and adolescents and intervene as early as possible [157]. Screening for obesity and MetS must be considered a public health priority, and it should be incorporated into many fields and occasions of pediatric clinical care, and obese individuals, particularly those also presenting MetS, should receive extra attention [161,162]. The data reported to date for obese children and adolescents show an odds ratio of 2.3–11.5 for developing T2D within 14–31 years [154,163], 2.0 for elevated carotid artery media thickness (a subclinical marker of the risk of CVD) within 14–27 years [163], and 14.6 for CVD within 24–31 years [154].

Talking with patients and their parents about how MetS in childhood increases the risk of future chronic disease and how this risk can be mitigated [159] may help motivate obese children and adolescents and their families [157].

Although MetS was originally described in adult patients, the cluster of cardiometabolic risk factors is increasingly present in obese children [94,164,165,166]. Autopsy studies in children reveal that atherosclerosis can be evident early in life and is associated with obesity, arterial hypertension (AH), high fasting triglycerides (TG), low high-density lipoprotein (HDL), cholesterol (HDL-C), and high fasting glucose [144,167,168]. The Bogalusa Heart Study [168] and Pathobiological Determinants of Atherosclerosis in Youth Study [169] strongly highlight that AH and dyslipidemia in children correlate with a higher prevalence of premature atherosclerosis. Therefore, the early identification of these risk factors is important in chronic disease prevention [170,171].

In predicting the future risk of CVD, it is important to recognize the clusters of risk factors associated with MetS [172,173]. The components of MetS are present together much more often than one would expect and are driven by similar underlying processes, including cellular dysfunction in adipocytes, myocytes, and hepatocytes, as well as oxidative stress and cellular inflammation [174,175].

MetS appears to be caused by a dysregulated cellular metabolism [174], leading to IR. Visceral adipocyte dysfunction appears to have a central role in MetS: central obesity releases both chemo-attractants contributing to macrophage infiltration and, at the same time side, cytokines, with an overall increase in systemic inflammation [175]. Visceral fat accumulation affects adipose tissue, stimulating the secretion of leptin and the hyposecretion of adiponectin [165,176,177]. Whereas high plasma leptin levels are related to the development of AH, hyperinsulinemia, and dyslipidemia [178], decreased adiponectin, an adipocytokine acting against the atherogenic, diabetic, and pro-inflammatory effect [179], is associated with a dysmetabolic state and appears to be in the causative pathway of IR [180] and also a cause of a greater release of free fatty acids (FFA) [181].

Furthermore, as obesity progresses, visceral obesity may lead to ectopic fat deposition in skeletal muscle, the liver, the heart, etc. (abnormal lipid partitioning) [182]. For example, Lee et al. showed that epicardial fat thickness (EFT) at the right ventricle, left ventricular apex, and atrium seems to correlate with visceral fat and acts as a cardiometabolic risk factor [183].

Hyperinsulinemia and IR are well-known risk factors for CVD, as well as AH, heart failure, and atherosclerosis [184,185], principally by increasing sodium reabsorption from the kidneys (due to hyperinsulinemia) and decreasing endothelial nitrous oxide production (due to IR) [186] but also acting synergistically with other mechanisms, such as the physical compression of the kidneys due to visceral fat [187], sympathetic nervous system activation, hyperleptinemia [188], hyperuricemia [189], and the activation of the renin–angiotensin–aldosterone system [190]. A positive familiar history of MetS may indicate a greater risk for IR and CVD [191,192].

In tissues, chronic obesity leads to the accumulation of FFAs in insulin-sensitive organs, such as the liver, adipose tissue, and skeletal muscle. This, together with chronic inflammation, results in impaired insulin signaling and selective IR. In the liver, the impaired insulin suppression of glucose production may lead to hyperglycemia, which can gradually progress to T2DM [193]. At the same time, the preserved insulin-mediated hepatic lipogenesis can lead to an increased release of FFAs and TG into the circulation. In peripheral tissues, high levels of FFA and TG alter mitochondrial functions, increasing oxidative stress, with a reduction in the ability of insulin to stimulate glucose transporters at the cell surface [174], also resulting in IR and, ultimately, T2D [194]. Further downstream effects include AH and reduced HDL-C levels, both of which are additional risk factors for CVD [157].

Thus, hepatic and adipose tissue dysfunction leads to a pattern of dyslipidemia, which is typical of MetS, i.e., elevated triglycerides, low HDL-C, relatively normal low-density lipoprotein cholesterol, and increased VLDL particles, all of which increase atherogenesis and the risk of CVD [195]. Finally, IR also stimulates the production of C-reactive protein, fibrinogen, and plasminogen activator inhibitor-1 (PAI-1), thus contributing to a proinflammatory and prothrombotic state [195]. This cytokine combination is proatherogenic, induces low-grade chronic inflammation and endothelial dysfunction, and further deteriorates IR, thus contributing to the atherogenic profile of MetS [195].

Puberty represents a particularly important time in exacerbating these effects, as body fat distribution, BP, and lipids are all affected by puberty. With puberty, sex hormones increase, leading to increased fat mass, androgen levels, and decreased sex hormone-binding globulin (SHBG) levels, all conditions promoting a “normal” IR [196,197]. In adolescent girls, elevated testosterone, low SHBG levels, and hyperandrogenism is associated with MetS [198,199]. HDL-C levels also decrease after puberty through the activity of the hepatic lipase [200] and because of the influence of sex hormones (particularly androgens) [201].

Furthermore, obesity and MetS can cause an increased metabolic and blood supply due to greater adipose tissue, which increases blood volume and cardiac preload. Vascular alterations, such as arterial stiffness and peripheral resistance, increase the afterload to the heart, favoring left ventricular (LV) hypertrophy, and LV diastolic dysfunction [202,203]. CIMT is a highly predictive marker of the progression of atherosclerosis in patients with MetS [204]; however, in very young children with MetS it may be normal [183].

Finally, another obesity-induced metabolic complication is hyperuricemia. Serum uric acid is typically associated with cardiovascular, renal, and metabolic diseases because of its pro-oxidant role inside the cell [205]. The link between uric acid levels and AH seems to be the strongest, with a high incidence of MetS and new-onset primary AH [189,206]. Children with AH and higher serum uric acid levels have a higher prevalence of obesity-related CVD [207]. It remains to be confirmed whether elevated uric acid levels actually cause CVD [208].

MAFLD, previously termed non-alcoholic fatty liver disease or NAFLD, is the main cause of liver disease worldwide [209] and has become the most common indication for liver transplantation [210]. This term encompasses a series of fatty liver disease states, relating to IR, starting with “benign” steatosis through to non-alcoholic steatohepatitis, a hepatic inflammatory condition leading to advanced fibrosis or cirrhosis [211]. The prevalence of MAFLD parallels that of obesity and MetS. It has been linked, in epidemiological studies, to the major features of MetS, such as IR, abdominal obesity, and T2DM [209,212].

In fact, MAFLD is considered by many as the hepatic expression of the syndrome. Its pathogenesis is related to hepatocyte fat accumulation in a genetically susceptible individuals, together with inflammatory adipokines, mitochondrial dysfunction, oxidative stress, and other factors which interplay to cause a gradual loss in normal hepatic architecture and function [213].

OSA is increasingly observed in children with MetS. Obesity can cause OSA, as the enlarged fatty soft tissues compress the airways, and the accumulated abdominal fat decreases the functional residual capacity and tidal volume of the lungs [214]. There is also growing evidence that other factors, such as glucose, insulin, and leptin abnormalities, are associated with more frequent apneic events, autonomic system dysfunction, and decreased chemosensitivity. In addition, it seems that OSA is not only a result but also a cause of MetS abnormalities in a bidirectional way [215].

PCOS is another condition that frequently coexists with MetS in obese adolescent girls, thus increasing the possibility of CVD and T2D [216]. It seems that IR and the resulting hyperinsulinemia augment both ovarian and adrenal androgen production while suppressing sex hormone-binding globulin, thereby increasing androgen bioavailability. Obesity may also contribute to hyperandrogenemia through enhanced androgen production by the increased fat mass and through abnormal adipokine levels [217].

### 3.9. Clinical Utility of the Metabolic Syndrome in Children and Adolescents with Overweight/Obesity: Facts or Myths?

In the last few decades, a growing interest in the obesity epidemic and its cardiometabolic comorbidities has been reported [136]. A strong association between pediatric obesity and cardiometabolic risk (CMR) factors, including IR, AH, dyslipidemia, and prediabetes/diabetes has been shown [136]. The presence of MetS during childhood is considered a useful tool for identifying high-CMR pediatric subjects.

Even though at least 11 different definitions of pediatric MetS (see previous paragraph) have been proposed, a relationship between MetS and OB/OW in developing NAFLD, subclinical atherosclerosis, and left ventricular hypertrophy (LVH) has been described.

In adults, a strong association between these three conditions and cardiovascular morbidity or mortality has been extensively analyzed [216,217,218]. Therefore, despite the absence of longitudinal studies, these entities have been considered as “surrogate” markers of CVR in children and adolescents with OB/OW.

### 3.10. NAFLD and MetS

The association between MetS and NAFLD has been widely analyzed in youths with OB/OW (see previous paragraph); however, whether the definition is more useful than its single components in identifying individuals with NAFLD has not yet been fully elucidated. As previously reported, young people with MetS have a significantly higher probability of developing NAFLD compared to subjects without MetS. This risk is not limited to individuals with complete MetS; one MetS component is sufficient to increase the odds of NAFLD in children and adolescents with obesity [212,219]. Among the single components, total or visceral obesity showed similar odds of NAFLD compared to MetS. In addition, high ALT levels (>25.8 IU/L in boys or 22.1 in girls) [220] were found to be useful in identifying individuals at the same risk of NAFLD as MetS [221]. Therefore, anthropometric or biochemical measures are simpler and more useful for identifying children and adolescents with obesity at risk of NAFLD compared to the complex and heterogeneous MetS definition.

### 3.11. Subclinical Atherosclerosis and MetS

In the past twenty years, IMT measurement has been considered a subclinical marker of vascular disease both in adults and children [222]. Currently, IMT in pediatric age is measured mainly in research and its application in clinical practice is still limited, due to the operator skill required, but IMT is considered a valid surrogate marker for CVR [223,224]. However, the impact of CMR factors on IMT remains debated: high WC [224], triglycerides to HDL ratio as surrogate of atherogenic dyslipidemia [225], high BMI [163], or impaired glucose tolerance [226] show similar abilities to detect increased IMT compared to MetS.

### 3.12. LVH and MetS

Data on the relationship between LVH and MetS remain limited and controversial. De Simone et al [217]. reported a close association between LVH and MetS in American Indian adolescents and young adults, independently of the relationship between individual components of the syndrome. On the other hand, Di Bonito et al. demonstrated that concentric LVH was associated with MetS, but this association was not higher compared to high BP alone [221]. These discrepancies could be related to several factors, including ethnic group, MetS definitions, or LVH.

However, it is interesting to underline that in young people with the MHO phenotype, LV mass levels or LVH prevalence were comparable to the parameters detected in subjects with the MUO phenotype [227,228]. These findings support the idea that obesity per se seems to be more influential than MetS in promoting LVH in children or adolescents with obesity.

In the last few years, the proposal to consider MetS as a tool to identify adults at high risk of CV events or T2D has been abandoned [173]; similarly, the role of MetS in clinical pediatric practice is still discussed. In this context, certain considerations should be taken into account:

(1) The literature evidence does not support a higher discriminant ability of MetS in identifying subjects at high-CMR, compared to its individual components;
(2) the largest number of reports on the relationship between MetS and subclinical marker of CVD risk are cross-sectional studies and a small sample size is usually considered;
(3) total body fat and visceral adiposity are the crucial players in developing CMR and are more important than all other comorbidities.

Considering these observations, the limited role of MetS in clinical practice should not be excluded in children or adolescents with OB/OW. As recently suggested by the American Academy of Pediatrics [161], it would be more useful to focus on the prevention and control of comorbidities by detecting a cluster of two components rather than the MetS entity according to different definitions.

### 3.13. Instability of MetS

The concept of MetS in children and adolescents is still not completely defined, as is often assumed, and likewise, its implications for clinical practice are still debated [229].

The lack of consensus regarding the definition of pediatric MetS is partially related to continuous improvement in our knowledge of the normal mechanisms underlying childhood and pubertal development. The abnormalities behind MetS develop progressively, following age-related changes linked to obesity, so that MetS cannot be diagnosed below the age of 6 years [95]. Therefore, differently from adult ages, where the main factors influencing the pathophysiological basis and main features of MetS are gender, ethnicity, and body composition, in children and adolescents, growth and puberty represent additional key factors that need to be factored in [230,231]. The presence of such developmental changes impairs the long-term reproducibility of the diagnosis of MetS, thus reducing its actual utility in childhood [87]. In this respect, previous authors have introduced and analyzed the concept of “stability” of the diagnosis of MetS both in children and in adults, defined as the persistence of a certain prevalence of diagnoses of MetS upon retesting over a short- or long-term follow-up period [232].

In the DESIR study on 4293 adults evaluated at baseline and after 3 years, more than one quarter of those patients classified as having MetS at baseline did not fulfil the criteria for MetS three years later or still had MetS but with different components [233].

Studies evaluating the instability of the diagnosis of MetS in children and adolescents have shown variable results. In a seminal paper by Goodman and co-workers, diagnoses of MetS in adolescents showed a rate of instability of around 50% over a follow-up of 3 years, regardless of the diagnostic criteria used [87]. Two more recent studies [234,235] involving adolescents followed-up for 2 years, revealed the instability of MetS in 33% [235] and 52–61.9% of the cases (depending upon the definition used) [234]. In another study [232] evaluating obese children and adolescents, the authors found a rate of the instability of diagnoses of MetS of about one third in a short-term cohort (220 subjects followed-up for 1–60 days) and of about 45% in a long-term cohort (146 subjects followed-up for 1.5–12.1 years), even when BMI remained above the 99th percentile. Relevant short- and long-term instability was also found in the identification of single components of MetS, up to 70% for triglycerides and up to 40% for impaired glucose tolerance and hypertension.

The main cause for short-term instability has to be sought in the variability of measurements. Given that the component criteria of MetS are continuous variables, several physiological factors, such as time of day, intercurrent diseases, or stressful events in general, and insufficient fasting may influence their recognition [232]. In keeping with this, in different studies [232,233], impaired fasting glucose has shown the highest rate of instability (up to 100%) compared to other components of MetS, attributable to the well-known high intra-individual variability of fasting glucose concentrations [236]. Intra-individual changes in weight-related variables, such as HDL-C and glucose concentrations, have also been demonstrated among adolescents who do not develop MetS over a follow-up of about 2 years [234].

Therefore, as already recommended for AH [237], testing for the other components of MetS needs to be standardized by establishing the precise circumstances of measurements, as well as repeated (confirmatory) testing.

In a more recent study [238], including a large cohort of adolescents with a follow-up of up to 10.4 years, the diagnosis of MetS showed a rate of instability ranging from 5.4 to 19.6%, based on the definition used. In particular, the pubertal group (11–14 years) had higher MetS instability than the late-pubertal group (15–18 years). On the other hand, in the study by Gustafson et al., MetS was found to be stable only among children with severe obesity who had entered puberty [232].

These data clearly suggest that, despite also being potentially related to the intrinsic variability of the measurement, pubertal changes and timing play a key role in the long-term instability of MetS. Indeed, puberty markedly influences adipose tissue deposition, lipids, and glucose metabolism, with a significant impact on health risk in obese subjects [239,240,241]. Therefore, the use of single cut-off values for metabolic parameters within the definition of MetS independently from measures of adiposity and/or physiological variations associated with age-, gender-, and pubertal status appears to be a critical point affecting the reliability of the diagnosis of MetS in adolescence.

A further factor which may affect subject clustering in childhood and adolescence is the choice of too liberal threshold values to define the abnormalities behind MetS [233]. In general, the pediatric definitions of MetS are adapted from those used for adults, mainly applying cut-off values for each component to children or adolescents [242]. This may in turn lead to an over-estimation of diagnoses of MetS in childhood/adolescence.

Studies assessing the persistence of MetS from adolescence to adulthood have revealed poor predictive value for adult MetS or cardiometabolic disease [163,238,243,244]. In the above-mentioned study by Ashgari et al. [238], the late-pubertal group, despite a lower percentage of incident MetS, showed a higher rate of persistence and of agreement with adult MetS, compared with the pubertal group.

In a study [243] involving 458 adolescents transitioning to adulthood, the rates of instability over a 10-year follow-up among cases diagnosed at baseline and after 4 and 8 years were 61.5%, 36.4%, and 25%, respectively. Instability did not correlate with changes in body weight; however, an increase in BMI was documented in 77% of unstable cases, indicating high instability of MetS during transition even in obese subjects to the point that MetS can remit even if BMI increases. In contrast with the results by Ashgari et al. [238], 56% of the incident cases developed by the end of follow-up, indicating that the increasing prevalence of MetS during transition is likely due to incident, rather than persistent cases. Interestingly, after stratification by pubertal status, the risk for incident MetS for each unit change in BMI was greater in the peripubertal group than the post-pubertal subjects, pointing toward the important role of weight gain during puberty in the pathogenesis of MetS.

Similarly, in a smaller study [235] on 73 overweight/obese adolescents of Latino origin and with a family history of T2D, the persistence of MetS during follow-up was associated with lower insulin sensitivity and lower disposition index, which estimates compensatory adaptation to IR, compared to subjects who never classified as MetS over a 2-year follow-up.

In order to overcome the risk of instability of the diagnoses of MetS in adolescence, some authors have suggested the use of continuous scores [172], which have been evaluated to reduce racial/ethnic discrepancies in adolescents [245], while others have pushed for an approach that simply focuses on cardiometabolic risk factor clustering.

Further efforts have to be made to improve the recognition of MetS during adolescence and transition.

## 4. Conclusions

Due to the increasing prevalence of obesity in childhood in recent decades, which has become a matter of concern for public health, the traditional approach to diagnosis, prevention and treatment should be improved. During childhood, excessive body fat increases the risk of obesity in adult life by five-fold and is associated with CMR complications, such as central obesity, AH, dyslipidemia, and impaired glucose metabolism.

The scientific literature reports diverse definitions of MetS at the pediatric age. Although there is an agreement on the distinctive features of MetS, no univocal international diagnostic criteria in a pediatric population exist. As a consequence of the lack of a unanimous definition of MetS, to date, its prevalence in childhood is not definite and the true value of the diagnosis of MetS in youth is not clear.

The definition of MetS in a pediatric population could allow pediatricians to promptly identify and treat children with an increased risk of future adverse outcomes. From an epidemiology point of view, the advanced identification of children with the greatest need for risk reduction could provide the opportunity to perform prospective studies with the aim to evaluate the efficacy of an early and intensive treatment of overweight.

Furthermore, in the last few years, the idea that MetS in adults is useful for identifying individuals with high CVR events or T2D has been abandoned.

Through critical discussion, this narrative review summarizes the pathogenesis and current diagnostic role of MetS in children and adolescents and its applicability in clinical practice in managing childhood obesity in the context of existing literature data.

Taken together, these data raise important concerns regarding the use of MetS in adolescence and its utility in predicting adult MetS, especially in the absence of significant weight gain in adolescence.

Perhaps it might be more useful to focus pediatricians’ attention on the prevention of childhood obesity and the control of comorbidities by detecting a cluster of two factors rather than all the components of MetS, taking into account that the distinction between obese phenotypes could provide more effective and targeted treatment for overweight.

In this regard, the Working Group of Childhood Obesity (WGChO) of the Italian Society for Pediatric Endocrinology and Diabetology (ISPED) is involved in promoting several campaigns and prevention strategies to raise public awareness on the importance of early identification and intervention in childhood obesity, as well as in the drafting of national guidelines.

## Figures and Tables

**Table 1:** Diagnostic criteria for Metabolic Syndrome (MetS).

**National Cholesterol Education Program (NCEP) Adult Treatment Panel (ATP) III** [86]	At least three of the following criteria: WC ≥ 102 cm in men and WC ≥ 88 in women IFG ≥ 100 mg/dL or hyperglycemia LDL-C ≥ 130 mg/dL TG ≥ 100 mg/dL in children aged 0–9 years and TG ≥ 130 mg/dL in those aged 10–19 years HDL-C < 40 mg/dL in men and <50 mg/dL in women
**International Diabetes Federation** [8]	WC ≥ 90th percentile for age and sex associated with at least 2 of the following: IFG ≥ 100 mg/dL TG ≥ 150 mg/dL HDL-C ≤ 40 mg/dL BP ≥ 130/85 mmHg
**IDEFICS Study** [91]	≥3 of the 4 following criteria: WC ≥ 90th percentile (monitoring level) or ≥95th percentile (action level) Systolic and/or diastolic BP ≥ 90th percentile (monitoring level) or ≥ 95th percentile (action level) TG ≥ 90th percentile (monitoring level) or ≥ 95th percentile (action level) or HDL-C ≤ 10^th^ percentile HOMA-IR or FG ≥ 90th percentile (monitoring level) or ≥ 95th percentile (action level)
**Cook et al.** [88]	≥3 of the 5 following criteria: WC ≥ 90th percentile BP ≥ 90th percentile TG ≥ 100 mg/dL HDL-C ≤ 40 mg/dL IFG (≥100 mg/dL)
**De Ferranti et al.** [90]	≥3 of the 5 following criteria: WC ≥ 75th percentile BP ≥ 90th percentile TG ≥ 100 mg/dL HDL-C ≤ 50 mg/dL IFG ≥ 110 mg/dL
**Zong et al.** [95]	WC ≥ 90th percentile for age and sex, associated with, at least, 2 or more of the following: IFG ≥100 mg/dL TG ≥ 130 mg/dL for 10–17-year-olds or ≥ 100 mg/dL for 6–9-year-olds HDL-C < 40 mg/dL Systolic or diastolic BP ≥ 90th percentile for sex, age and height

IDEFICS: the identification and prevention of dietary- and lifestyle-induced health effects in children and infants. WC: waist circumference; IFG: impaired fasting glucose; TG: triglycerides; HDL-C: high-density-lipoprotein cholesterol; BP: blood pressure; HOMA-IR: homeostatic model assessment for insulin resistance.

**Table 2:** Pooled prevalence of MetS according to different definitions from the two most recent systematic reviews.

	Study Collection	Population	Countries	IDF	Modified ATP III	de Ferranti et al. [90].	Cook et al. [88].
Bitew et al. 2020 [110]	until July, 2020	3906 youths with OW/OB; age range 5–20 years	Europe (Serbia) Asia (Myanmar, Iran, Philippines, Thailand) Africa (Tunisia, Egypt) America Latina (Brazil Bolivia Peru Argentina Mexico)	24.1% (95% CI 16.90, 31.29) 14 studies	36.51% (95% CI −1.76, 74.78) 8 studies	56.32% (95% CI 22.34, 90.29) 2 studies	NR
Obita et al. 2022 [111]	from January 2010 to Feb 2022	45,889 youths with OB; age range 6–19 years	Europe (Greece and Turkey), South America (Colombia, Mexico, and Chile), Asia (China, Korea, and the United Arab Emirates), and Africa (including Tunisia)	Overall, 26.1% (range 7.7–72.8) 10 studies Europe 20.4 (7.7–33) South America 72.8% (Colombia) Asia 26.15 (16.8–41.2) Africa 14.3%	Not reported	Not reported	33.3% (26.3%-40.3%) (Chile–Mexico), 2 studies

## Data Availability

No new data were created or analyzed in this study. Data sharing is not applicable to this article.
